# Social determinants of health Z-code documentation practices in mental health settings: a scoping review

**DOI:** 10.1093/haschl/qxae046

**Published:** 2024-04-12

**Authors:** Rachele M Hendricks-Sturrup, Sandra E Yankah, Christine Y Lu

**Affiliations:** Duke-Robert J. Margolis, MD, Institute for Health Policy, Washington, DC 20004, United States; Duke-Robert J. Margolis, MD, Institute for Health Policy, Washington, DC 20004, United States; Department of Population Medicine, Harvard Pilgrim Health Care Institute and Harvard Medical School, Boston, MA 02215, United States; Faculty of Medicine and Health, Kolling Institute, The University of Sydney and the Northern Sydney Local Health District, St Leonards, NSW 2065, Australia; Faculty of Medicine and Health, School of Pharmacy, The University of Sydney, Camperdown, NSW 2050, Australia

**Keywords:** social determinants of health, electronic health record, mental health, Z codes, health policy

## Abstract

Mental health remains an urgent global priority, alongside efforts to address underlying social determinants of health (SDoH) that contribute to the onset or exacerbate mental illness. SDoH factors can be captured in the form of International Classification of Disease, Tenth Revision, Clinical Modification (ICD-10-CM), SDoH Z codes. In this scoping review, we describe current SDoH Z-code documentation practices, with a focus on mental health care contexts. Among 2 743 061 374 health care encounters noted across 12 studies in the United States, SDoH Z-code documentation rates ranged from 0.5% to 2.4%. Documentation often involved patients under 64 years of age who are publicly insured and experience comorbidities, including depression, bipolar disorder and schizophrenia, chronic pulmonary disease, and substance abuse disorders. Documentation varied across hospital types, number of beds per facility, patient race/ethnicity, and geographic region. Variation was observed regarding patient sex/gender, although SDoH Z codes were more frequently documented for males. Documentation was most observed in government, nonfederal, and private not-for-profit hospitals. From these insights, we offer policy and practice recommendations, as well as considerations for patient data privacy, security, and confidentiality, to incentivize more routine documentation of Z codes to better assist patients with complex mental health care needs.

## Introduction

Mental health disorders have been and continue to be in the wake of COVID-19 a major global health priority, as they affect 1 in 8 individuals annually, contributing to numerous deleterious physical health effects.^1^ Decades of research have demonstrated a bidirectional relationship between common psychiatric conditions (eg, depression, anxiety) and the development and exacerbation of several chronic health conditions (eg, cardiovascular disease, diabetes, and chronic pain), highlighting the inextricable link between mental and physical health.^2-4^ In addition to compounding health risk, outcomes associated with unresolved psychiatric symptoms include increased health care expenditures and reduced educational and occupational attainment, augmenting the economic effect of these conditions.^5^ The Association of American Medical Colleges projects that, within a few years, the United States will be short between 14 280 and 31 109 psychiatrists.^6^ This anticipated shortage poses a significant challenge to the practice of medicine overall, and is a concern acknowledged by the American Medical Association. Current and anticipated shortages of mental health care providers pose significant challenges to the health system writ large, prompting immediate action among policymakers to enact measures intended to provide coordinated equitable care and support for patients, families, and clinicians.^7^ However, such measures often require care coordination and communication between the health system and local community-based entities that have the capacity, resources, bandwidth, and tools to address social determinants of health (SDoH) factors that contribute to the onset of or that exacerbate mental illness.

Today, pathways exist to document SDoH factors within electronic health records (EHRs; ie, clinical notes and International Classification of Diseases, Tenth Revision, Clinical Modification [ICD-10-CM], documentation). Z codes ranging from Z55 to Z65 are used to document SDoH data (eg, housing, food insecurity, transportation, etc), and may contextualize a patient's health-related circumstances and/or experiences (eg, financial, logistical, and social needs) at the clinical encounter.^8,9^

Reflecting on the importance of holistic care approaches that take all of these factors into account, clinicians are increasingly encouraged to document ICD-10 Z codes that reflect SDOH factors across 10 discrete categories: Z55 (education and literacy), Z56 (employment), Z57 (occupational exposure), Z58 (physical environment), Z59 (housing and economic circumstances), Z60 (social environment), Z62 (upbringing), Z63 (primary support group), Z64 (psychosocial circumstances), and Z65 (other psychosocial circumstances).^10^ Although Z61 (loss of love relationship in childhood) is not highlighted as an SDoH Z code by the Centers for Medicare and Medicaid Services (CMS), our impression is that it can be subjectively considered as a circumstantial and contextual SDoH and diagnostic factor during a health care encounter.^10^

Health system stakeholders, including health care providers, have discussed the importance of SDoH Z codes, as they could indicate the risk of adverse treatment effects or poor treatment outcomes.^11,12^ Likewise, payers and health care providers have described how SDoH Z codes allow them to collect data that are needed to identify and/or provide solutions, services, and/or supports that more closely align with patients' needs.^13-17^ This would include medical-legal partnerships that integrate legal experts into clinical settings to assist patients and their families with addressing SDoH needs through civil legal procedures.^17,18^ Some studies to date have assessed overall SDoH Z-code utilization in general (vs specialty) practice settings.^19-21^

Current SDoH Z-code documentation practices in mental health care contexts have yet to be summarized in the literature. In this technical review of the literature, we describe SDoH Z-code documentation practices currently reported in the literature, with a focus on mental health care contexts to provide policy and practice recommendations to drive stronger patient-level support in mental health care.

## Data and methods

### Literature search

For this study, we followed the Preferred Reporting Items for Systematic Reviews and Meta-Analyses extension for Scoping Reviews (PRISMA-ScR) checklist (see Table S1). We conducted a scoping review of the most recent studies (<5 years, 2019–2023) reported in peer-reviewed literature on Z-code documentation in relation to mental health care delivered within the United States (inclusion criteria). Our search was limited to studies published within the past 5 years for the following reasons: (1) although Z codes were first introduced in 2015/2016, clinical documentation of SDoH Z codes has been reportedly slow and low, likely due to practice-level complexities that have been discussed in the literature^22,23^; (2) health disparities due to SDoH-related factors became more apparent during the COVID-19 pandemic; and (3) mental illness, including substance abuse, became a global public health crisis and concern following the COVID-19 pandemic.^9,24^ A search was conducted in March 2024 for papers indexed in PubMed, PsycINFO, Scopus, and Web of Science, or searchable in Google Scholar. The following search terms were used alongside Boolean search logic: Z code, Z-code, psychiatry, mental (ie, [“Z code” OR Z-code] AND [psychiatry OR mental]). Access to full-text articles was made available through the Duke University Library. Papers published outside of this time frame, not published in the English language, and that did not present evidence on SDoH Z-code utilization in US mental health care settings/contexts were excluded from the review.

### Data extraction and analysis

One author (R.M.H.-S.) extracted the following information from the included articles: author(s), publication year, Z-code variables of interest related to SDoH, and key findings relevant to psychiatric and behavioral health care. Two authors (S.Y. and C.Y.L.), reviewed the information extracted, and the research team used constant comparative and group-level analysis against the original papers to review, discuss, and validate findings until strong (>95%) agreement was reached among all 3 authors.

## Results

Upon retrieving results in PubMed (11 results), PsycINFO (19 results), Scopus (91 results), Web of Science (24 results), and Google Scholar (782 results), 12 studies met our inclusion criteria and were included in our final analysis (see Table 1 and Figure S1). Among 2 743 061 374 health care encounters reported across the 12 studies, SDoH Z-code documentation rate ranged from 0.5% to 2.4%, notwithstanding 1 study^33^ that analyzed an intentional sample of 2080 patients with documented homelessness and opioid use disorder without reporting an overall SDoH Z-code reporting rate.

**Table 1.** Summary of studies reporting International Classification of Diseases, Tenth Revision, Clinical Modification, social determinants of health (SDoH) Z-code documentation and key findings relevant to mental health care.

Author (year)	SDoH Z code(s) screened	Percentage of cases with a documented SDoH Z code	Key findings relevant to mental health care
Gibbons et al (2023)^25^	Z55–Z65	Among 3.5 million Medicaid claims, 0.8% had a documented Z code, 1.6% had 1 or more Z-code diagnoses. Among 9.4 million commercial claims, 0.5% of the total had a documented Z code. A total of 1.0% had 1 or more Z-code diagnoses.	Higher usage of Z codes was observed in specific mental health care settings for Medicaid patients. Common Z codes for Medicaid cases involved low-income, parent–child conflict, unemployment; for commercial: relationship issues, parent–child conflict. Z-code claim frequencies were similar across provider types. Patient demographics showed distinct age and gender patterns. The proportion of females was notably high for certain SDoH Z codes, such as Z64 (psychosocial circumstances) and Z63 (primary support group). Additionally, among Medicaid patients, the mean ages for those with the most frequently documented SDoH Z-code domains varied, ranging from 16 years for Z62 (upbringing) to 39 years for Z56 (employment).
Yoder et al (2023)^26^	Z55–Z65	Among 73 443 patient subjects, 1.6% SDoH codes were documented during critical care services; 1.4% were documented 1 year before; and 1710 (2.3%) were documented 1 year after critical care services.	Among patient subjects across health care organizations, 1.6% had SDoH codes during critical care services. There was a significant positive association of SDoH codes with mental, behavioral, and neurodevelopmental disorders and a higher prevalence of SDoH codes among Black patients, female patients, and variations by age and region.
McQuistion et al (2023)^27^	Z55–Z60, Z62–Z65	Among 6 266 285 inpatient pediatric visits discharges in 2016 and 5 902 538 discharges in 2019, SDoH code documentation was present in 1.9% of visits in 2019, and in 1.4% of visits in 2016.	Increase in SDoH documentation in pediatric inpatient visits from 2016 to 2019. SDoH Z codes were most frequently observed in mental health–related admissions. Adolescents and Native American pediatric patients had the highest rates. There were significant increases across hospital types and regions.
Molina et al (2022)^28^	Unspecified	Among 35 807 950 total adult and pediatric emergency department visits, 1.21% (weighted) had at least 1 documented SDoH Z code.	Highest odds of SDoH Z codes were in mental health diagnoses. There were variations in documentation across hospital types. Patient demographics varied by age, gender, and insurance coverage.
Bensken et al (2022)^29^	Z55–Z65	Among 4 477 772 adult patients, 1.03% had documented Z codes.	Patients with Z codes showed greater negative health events like depression and substance abuse. Patients with Z codes tended to be younger, White, and covered by Medicaid or self-pay.
Rollings et al (2022)^30^	Housing-related Z codes (eg, Z59.0)	Among 87 348 604 hospitalization cases, 1.08% hospitalizations had documented Z codes for housing instability.	Common reasons: mental disorders, injury, circulatory system diseases. Patients with housing instability were often homeless, male, Black, and publicly insured.
Liss et al (2022)^31^	Z55–Z65, Z77, Z91.120	Among 29 500 000 adult patients, 1.8% had a documented Z code.	Increased odds of Z codes in cases of depression and chronic pulmonary disease. Common Z codes: community/social factors, environmental factors, socioeconomic factors. Patients with Z codes tended to be older, female, and insured through Medicare Advantage.
Yang et al (2022)^32^	Z55–Z65	Among 1 190 531 total adult patients, 1.23% had documented Z codes.	Frequent Z-code categories: housing, upbringing, family circumstances. Higher odds of Z codes among patients negative for COVID-19. Facilities with Z codes were larger and teaching hospitals. Black or Hispanic patients had lower odds of receiving Z codes.
Ali et al (2021)^33^	Z59.0	Among 70 million unique patients, a sample of 2080 patients with documented homelessness and opioid use disorder (vs those without documented homelessness and opioid use disorder) was analyzed (ie, Z-code rate not reported).	Analysis of patients with homelessness and opioid use disorder. Higher prevalence rates for bipolar disorder and schizophrenia. Patients with Z59.0 code tended to be male, White/not Hispanic, and publicly insured.
Bensken et al (2021)^34^	Z55–Z65	Among 17 978 754 hospitalizations, 2.4% had at least 1 documented Z code.	Common Z-code needs: housing, socioeconomic status, family, employment. High rates of alcohol abuse, drug abuse, psychoses, and depression among patients with Z codes. Patients with Z codes tended to be male and publicly insured.
Truong et al (2020)^35^	Z55–Z65	Among 14 289 644 inpatient hospitalizations, 1.9% had documented SDoH Z codes.	Higher proportion of Z codes for mental health and substance use disorders. Hospitals with Z codes were larger, private not-for-profit, and urban teaching. Patients with Z codes were more likely to be male, Black, aged 18–64, Medicaid or no charge, and low income.
Weeks et al (2020)^36^	Z55–Z57, Z59–Z60, Z62–Z63	Among 30 523 773 Medicare beneficiaries, 0.96% had a documented Z code.	Z codes most documented among beneficiaries with alcoholism, drug disorders, psychotic disorders. Beneficiaries with dementia, substance use, psychotic disorders, mood and anxiety disorders showed specific social, employment, and housing challenges. Beneficiaries with Z codes tended to be younger, male, Black, with lower incomes and higher condition scores.

Across 12 studies, health system encounters with a documented SDoH Z code often involved individuals/patients under 64 years of age. Most frequently documented SDoH Z codes involved issues around housing and economic circumstances, upbringing, primary support group, and social environment. Patients with documented Z codes were more likely to be publicly insured (ie, Medicaid). Last, patients with documented Z codes had higher odds of being publicly insured and experiencing comorbidities, including depression, bipolar disorder and schizophrenia, chronic pulmonary disease, and substance abuse disorders.

Z-code documentation varied across hospital types, although 3 studies^28,32,35^ reported teaching hospitals as having a higher proportion and/or odds of having a documented SDoH Z code. Among studies reporting hospital/health center capacity (ie, number of beds) Z-code documentation also varied based on the number of beds per facility (ie, small, medium, and large).^27^ However, 1 study^32^ found that facilities with documented Z codes were more likely to have higher capacity or a greater number of beds and another^27^ reported that freestanding children's hospitals (vs non-freestanding) had slightly lower odds of SDoH Z-code documentation. Variation was observed regarding patient sex/gender, although SDoH Z codes were more frequently documented for males.^25,26,28-36^

Among studies reporting patient race/ethnicity, SDoH Z-code documentation variation was observed for adult and pediatric patients reported as Black, White, Native American, Hispanic, and Other.^26,27,29,30,32,33,35,36^ Among studies reporting trends based on geographic region, SDoH documentation was observed across all census regions (South, Midwest, Northeast, West) regardless of income quartile based on zip code.^26,27,28^ Two studies showed that private (not-for-profit or investor-owned) hospitals had a lower proportion and odds of SDoH documentation than government, nonfederal hospitals.^27,28^ One study showed that larger, private not-for-profit hospitals had at least 1 documented SDoH Z code.^35^

## Discussion

Social determinants of health Z codes can be used to document and track a range of social and environmental risk factors, with direct implications for treatment outcomes in psychiatric patient populations. Yet, despite expert consensus on the health relevance and importance of considering environmental variables on mental health outcomes that may or may not be genetically associated,^13-15,37-46^ to date, our findings show that SDoH Z codes are documented at a very low rate in health care practice settings that provide mental health care. This finding is slightly consistent with prior work indicating a generally low rate of SDoH documentation using International Classification of Diseases, Ninth Revision (ICD-9), coding procedures to assess associations between adverse SDoH and mental health risk in the form of suicide.^47^ Practical complexities that have been discussed among empirical studies include misunderstandings around who within a clinical setting can or should document a patient's social needs, a general lack of standard operating procedures for routine Z-code documentation, a lack of familiarity with SDoH Z codes among health administrators, and a possible likelihood that providers (vs billing and compliance staff) may be less aware of SDoH Z codes.^8,22^ The potentially subjective nature of many Z codes (ie, subjectivity of problems relating to loneliness/isolation, upbringing, housing and economic circumstances, etc) adds to this complexity. Future empirical work should explore this further.

Our findings are helpful to inform the clinical and real-world evidence policy communities on the current use and potential of SDoH Z-code documentation if more systematically and broadly used in mental health care practice. First, current practices of SDoH Z-code documentation may inspire further generation of evidence on contextual factors associated with psychiatric patient hospital admissions and outcomes. Second, although SDoH Z-code documentation is presently low, as described in our findings, there is large health system potential to further modernize their data-collection practices with the intent to more effectively connect patients to social and personalized health services during mental health crises. Last, but not least, SDoH Z codes may inspire ideas to contribute new data or data proxies (ie, time to social and/or personalized health service referral) for monitoring patient outcomes longitudinally in partnership with health system stakeholders that invest in the development and/or refinement of delivering SDoH-focused services at local community levels (ie, patients and their caregivers, payers, clinicians, health systems, and policymakers).^48^

We provide policy and practice recommendations below, based on our findings, for incentivizing routine documentation of Z codes in mental health care to address SDoH challenges and complexities. We also discuss patient-level considerations, particularly with respect to data privacy and confidentiality. Last, we discuss limitations to our present findings to suggest areas for further research.

### Payer incentive strategies

Presently, the use of Z codes for documenting or tracking SDoH risk dimensions may not enhance reimbursement or lead to patient referrals that significantly affect patient outcomes. Although providers may assess or address these factors in care settings, and in some cases document them in other portions of a patient's chart, the tangible benefit associated with Z-code documentation is unclear in the literature and should be addressed.

Evidence on the clinical, personal, and/or economic utility of Z-code utilization may open new avenues for payers to design payment models and benefit plans that more appropriately risk-adjust for SDoH, as well as segments and benchmark or target contract pricing in value-based purchasing arrangements. Payers should, therefore, consider 5 strategies to help standardize and incentivize routine Z-code use among mental health care providers, as follows:

- Create reimbursement mechanisms that encourage Z-code utilization across psychiatric care settings;
- Develop system-level processes and procedures that support the systematic collection of Z codes across emergency and non-emergency psychiatric care settings;
- Address structural challenges (ie, limited access to EHR infrastructure) associated with the use of Z codes in nontraditional clinical settings that may serve high-risk populations (ie, homeless populations with psychiatric illness);
- Promote the use of empirically validated methods of assessing dimensions captured by Z codes across psychiatric treatment;
- Pursue partnerships with practitioner educational programs to facilitate sharing of up-to-date information on the clinical utility of SDoH Z codes for treatment planning and standardized progress monitoring.

### Clinician education incentive strategies

The extent to which capturing SDoH Z codes has been incorporated into mental health education varies significantly across various disciplines within the mental health domain (eg, psychiatrist, psychologist, licensed professional counselors) and institution-specific training programs. Despite this variability, consensus on the importance of considering SDoH factors in the assessment and treatment of mental health conditions is growing. For example, the Association of American Medical Colleges developed a set of competencies for addressing SDoH in medical education, including in psychiatry training. These competencies include understanding the impact of SDoH on mental health outcomes, and identifying and addressing patients' social needs, and working collaboratively with other health care professionals to address SDoH. Similarly, the Accreditation Council for Graduate Medical Education has incorporated SdoH into the requirements for psychiatry residency training, including the ability to identify and address patients' social needs, and the ability to work collaboratively with other health care professionals to address SdoH.

### Health system strategies

Z-code documentation could provide a more streamlined mechanism for recording SDoH data, compared with typical unstructured documentation practices, within and across health systems offering mental health care services. For instance, 1 study found that 84% of physicians reported that their practices recorded patients' SDoH information (ie, employment status, educational level, and other health-related social needs) within their respective EHR systems.^49^ Overall, recording patients' SDoH within the EHR was common and occurred across a wide variety of practices, suggesting that practitioners are already collecting patient SDoH data using formats other than Z codes (eg, unstructured narrative formats in clinical notes). Improving the rate of Z-code documentation of SDoH has the propensity to streamline the documentation process, improve the accuracy and breadth of current SDOH documentation, and facilitate improved interoperability for individualized patient care.^49^ Accordingly, further work should explore whether documentation of SDoH characteristics using Z codes could either contribute to or alleviate EHR documentation burden and improve interoperability across practice settings. In any case, meaningful SDoH documentation would require practice changes that accommodate documentation within the clinical workflow, standardized assessment tools and methods, care coordination resources, and EHR capability within and across health systems offering mental health care services.

Health systems should consider the following strategies to increase the rates of Z-code utilization in routine psychiatric practice: (1) shift organizational culture by emphasizing biopsychosocial models of assessment and intervention as the standard protocol across practice settings and patient populations to reduce the burden on service providers and embed the assessment and treatment of these dimensions into routine clinical practice; (2) create more robust social support teams, including expanded teams of social workers to ensure that identification of social risk factors does not result in additional provider or system level burden; (3) pursue direct partnerships with state and federal social welfare agencies and community-based organizations to create a network of support for patients and facilitate quick responses for acute patient needs; and (4) integrate the collection and tracking of dimensions captured by Z codes into health system quality-improvement efforts and financial goals.

### Policy incentive strategies

In 2016, the Centers for Medicare and Medicaid Innovation (CMMI) proposed the Accountable Health Communities (AHC) initiative. The CMMI-AHC program promoted screening and documentation of unmet health and social needs, directly assessed, and provided targeted interventions among a subset of Medicaid and Medicare beneficiaries with disproportionally high rates of health care cost and utilization. The program has also facilitated partnerships with community-based organizations that connect beneficiaries to local services with capabilities to provide time-sensitive intervention and resources for acute needs (eg, housing, food insecurity, transportation needs). As part of the program, service cost-savings associated CMMI-AHC created a screening tool that prioritizes 5 core domains focused on health-related social needs, including (1) housing, (2) food, (3) transportation, (4) utilities, and (5) interpersonal safety. The CMMI-AHC tool also includes a supplemental questionnaire that assesses a wide range of SDoH. Resources like the CMMI-ACH tool have the propensity to standardized the assessment of broader patient-level characteristics with implications for care outcomes in psychiatric settings. The CMMI has also highlighted the tool's ability to be integrated in psychiatric care settings to inform public and private payers and assist in the documentation of SDoH with Z codes during clinical encounters. When used in conjunction with standardized capture methods facilitated by Z codes within a patient’s EHR, tools like the AHC questionnaire can also be integrated into standard protocols for treatment planning and community referrals to services. This could include, but not be limited to, referrals to medical legal partnership programs that work closely with patients and their families to obtain needed services relate to SDoH and/or primary health care providers with integrated behavioral health services.^50^

### Considerations for patient data privacy, security, and confidentiality

The literature is presently scant on the topic of patient privacy and confidentiality concerns and SdoH Z-code documentation in health care settings in general, including mental health care settings,^51^ although we believe concerns about any SDoH data documentation might align with generalizable concerns about EHR data privacy and security (eg, unauthorized access, data breaches, insider threats, improper data sharing, re-identification risks, and inadequate data retention and disposal practices). Typically, sharing social and mental health data mandates explicit patient authorization, unless such authorization is waived by a privacy board, Institutional Review Board (IRB), or social service agency. While research professionals and health care systems are obligated to uphold compliance with all foundational state and federal laws, fostering trust among patient communities may necessitate the implementation of enhanced protocols for access control, transparency, and consent management.^51^ Further work should explore this topic as a mechanism to ensure health system and provider trustworthiness, especially given the exceptionally sensitive nature of both patient- and population-level social data alongside mental health care information. Additionally, future work should examine strategies for mitigating privacy risks, enhancing data security measures, and identifying/refining documentation practices that uphold patient confidentiality while effectively leveraging SDoH data across mental health care contexts.^51^

### Limitations

There are some limitations to our scoping review. First, our literature search was conducted in PubMed and Google Scholar only; it is possible that other potentially relevant publications in unsearched databases might have been missed. We included only fully published articles; studies may continue to be underway, given that 1 relevant study has been published recently as a conference abstract vs as an original report in peer-reviewed literature.^52^ To address these limitations, it will be important to support work that attempts to document and report health system practices, policies, and procedures that might affect Z-code documentation in medical practice generally and especially in mental health care settings.

## Conclusion

Social determinants of health Z codes are rarely documented in both public and private health care encounters. Yet, based on current documentation practices captured in this technical review, there are substantial opportunities to systematically documenting and tracking these dimensions across mental health care contexts. Although increased Z-code utilization rates may require robust efforts to shift standard organizational practice and reimbursement models, incentivizing routine documentation of Z codes in mental health care contexts holds great potential to effectively address unique SDoH challenges for patients with complex, yet sensitive mental health care needs.

## Supplementary Material

qxae046_Supplementary_Data
