# Supplementary figures and images for: Social determinants of health Z-code documentation practices in mental health settings: a scoping review
Source: Health Aff Sch. 2024 Apr 12;2(4):qxae046. doi: 10.1093/haschl/qxae046 (PMC11050653; doi:10.1093/haschl/qxae046)

Figure S1. PRISMA Flow Diagram

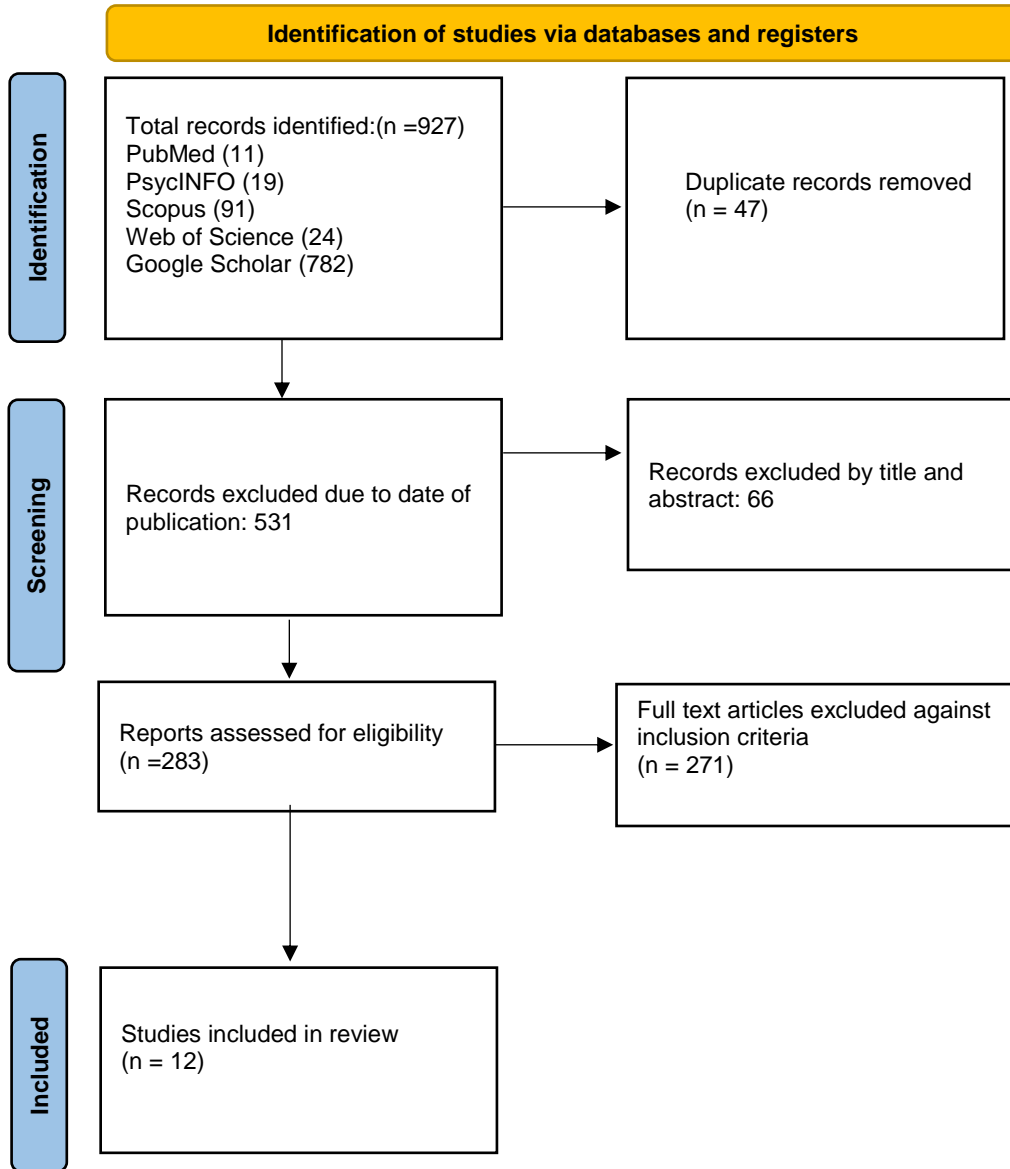

Supplement: qxae046_Supplementary_Data [file qxae046_supplementary_data.zip › Supplement Figure 1.pdf]
